# {2,2′-[3-Aza­pentane-1,5-diylbis(nitrilo­methyl­idyne)]dipyrrol-1-yl}(4-methyl­pyridine)cobalt(III) tetra­phenyl­borate

**DOI:** 10.1107/S1600536810006112

**Published:** 2010-02-24

**Authors:** Soraia Meghdadi, Mehdi Amirnasr, Kurt Mereiter, Mahmood Karimi Abdolmaleki

**Affiliations:** a Department of Chemistry, Isfahan University of Technology, Isfahan 84156 83111, Iran; b Faculty of Chemistry, Vienna University of Technology, Getreidemarkt 9/164SC, A 1060 Vienna, Austria

## Abstract

The title compound, [Co(C_14_H_17_N_5_)(C_6_H_7_N)](C_24_H_20_B) or [Co{(pyrrole)_2_dien}(4-Mepy)]BPh_4_ where (pyrrole)_2_dien is 2,2′-[(3-aza­pentane-1,5-diylbis(nitrilo­methyl­idyne)]dipyrrole and 4-Mepy is 4-methyl­pyridine, contains a penta­dentate (pyrrole)_2_dien ligand furnishing an N_5_ set, such that two of the pyrrole N atoms and two of the dien N atoms occupy the equatorial positions while one of the imine N atoms of the (pyrrole)_2_dien ligand occupies the axial position. The 4-methyl­pyridine ligand occupies an axial position *trans* to one of the imine N atoms of the penta­dentate ligand. In the observed conformation of the penta­dentate ligand, the pyrrole rings attain asymmetrical positions owing to the structural demands. The geometry of the resulting CoN_6_ coordination can be described as distorted octa­hedral.

## Related literature

For general background to the applications of transition metal–Schiff base complexes, see: Nishinaga & Tomita (1980 ▶); Speiser & Stahl (1995 ▶); Miodragović *et al.* (2006 ▶); Amirnasr *et al.* (2006 ▶); Morshedi *et al.* (2006 ▶); Meghdadi *et al.* (2007 ▶, 2008 ▶); Park *et al.* (1998 ▶); Mishra *et al.* (2008 ▶). For the synthesis of the ligand, see: Kwiatkowski *et al.* (1993 ▶). For related structures, see: Meghdadi *et al.* (2007 ▶, 2008 ▶).
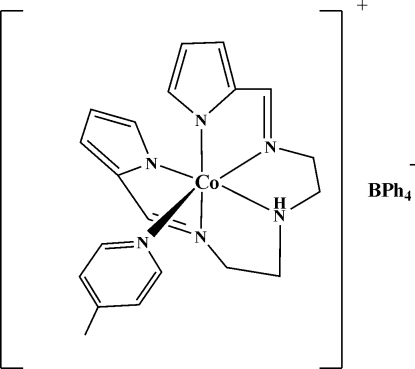


## Experimental

### 

#### Crystal data

[Co(C_14_H_17_N_5_)(C_6_H_7_N)](C_24_H_20_B)
*M* *_r_* = 726.59
Monoclinic, 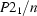

*a* = 11.0332 (16) Å
*b* = 19.559 (3) Å
*c* = 17.138 (3) Å
β = 92.164 (2)°
*V* = 3695.7 (9) Å^3^
*Z* = 4
Mo *K*α radiation
μ = 0.51 mm^−1^
*T* = 100 K
0.52 × 0.46 × 0.35 mm

#### Data collection

Bruker SMART APEX CCD diffractometer
Absorption correction: multi-scan (*SADABS*; Bruker, 2003 ▶) *T*_min_ = 0.71, *T*_max_ = 0.84
66110 measured reflections
10797 independent reflections
8767 reflections with *I* > 2σ(*I*)
*R*_int_ = 0.036

#### Refinement

*R*[*F* ^2^ > 2σ(*F* ^2^)] = 0.038
*wR*(*F* ^2^) = 0.104
*S* = 1.07
10797 reflections
474 parameters
H atoms treated by a mixture of independent and constrained refinement
Δρ_max_ = 0.57 e Å^−3^
Δρ_min_ = −0.22 e Å^−3^

### 

Data collection: *SMART* (Bruker, 2003 ▶); cell refinement: *SAINT* (Bruker, 2003 ▶); data reduction: *SAINT* and *XPREP* (Bruker, 2003 ▶); program(s) used to solve structure: *SHELXS97* (Sheldrick, 2008 ▶); program(s) used to refine structure: *SHELXL97* (Sheldrick, 2008 ▶); molecular graphics: *SHELXTL* (Sheldrick, 2008 ▶); software used to prepare material for publication: *SHELXTL* .

## Supplementary Material

Crystal structure: contains datablocks I, New_Global_Publ_Block. DOI: 10.1107/S1600536810006112/dn2536sup1.cif

Structure factors: contains datablocks I. DOI: 10.1107/S1600536810006112/dn2536Isup2.hkl

Additional supplementary materials:  crystallographic information; 3D view; checkCIF report

## Figures and Tables

**Fig. 1.**
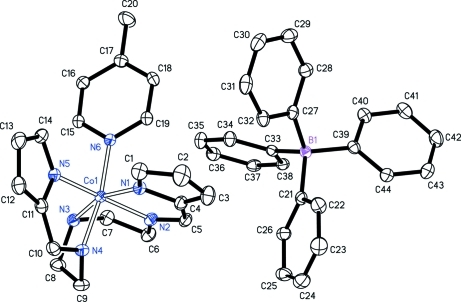
Molecular view of [Co{(pyrrole)2dien}(4-Mepy)]+ , with the atom labeling scheme. Displacement ellipsoids are drawn at 50% probability level. H atoms omitted for clarity.

## supplementary crystallographic information

### Comment

Transition metal Schiff-base complexes have been extensively studied due to their applications, e.g., their ability to reversibly bind oxygen, and their use in catalysis for oxygenation and oxidation reactions of organic compounds (Nishinaga *et al.* 1980; Speiser *et al.*, 1995; Park *et al.*, 1998). Among these metal complexes, cobalt(III) Schiff base complexes with two amines in axial positions have attracted considerable interest due to their ability as antimicrobial agents (Miodragović *et al.*, 2006; Mishra *et al.*, 2008). Transition metal complexes with pentadentate ligands show interesting structural features and have also been playing an important role in the development of coordination chemistry (Amirnasr *et al.*, 2006; Morshedi *et al.*, 2006; Meghdadi *et al.*, 2007; Meghdadi *et al.*, 2008) . In this context, we herein report the synthesis and structure of the title compound, [Co{(pyrrole)_2_dien}(4-Mepy)]BPh_4_, (I), and make a brief comparison with reported structures.

The environment surrounding Co^III^ in (I) is distorted octahedral (Fig. 1), in which the three N atoms of the Schiff base ligand are arranged in facial positions. The geometric parameters are listed below in the supplementary materials. The two chelate bite angles (81.38 (5)°, 82.26 (5)°) formed by the two imine-N and the secondary amine-N of the Schiff base are similar. The five-membered chelate rings formed by the pyrrole-N and the imine-N atoms have almost identical bite angles (N1—Co1—N2 82.11 (5)°; N4—Co1—N5 82.22 (5)°). The three *trans* angles, (N2—Co1—N5 170.28 (5)°; N4—Co1—N6 170.80 (5)°, N1—Co1—N3 161.96 (5)°) deviate significantly from ideal. The Co—*N*(pyrrole), Co—*N*(imine), Co—N(secondary amine) and CoN-(4-methylpyridine) bond lengths are comparable with the bond lengths observed in related Co(III) complexes (Meghdadi *et al.*, 2008). The conformation adopted by the (pyrrole)_2_dien in (I) is different from that of (Me-sal)_2_dpt in [Co{(Me-sal)_2_dpt}(py)]PF_6_ (Meghdadi *et al.*, 2007). While the three donor N atoms of (Me-sal)_2_dpt occupy three meridional sites and the two phenolate-O atoms are *trans* to each other, the three N atoms of (pyrrole)_2_dien ligand are arranged in facial positions and the two pyrrole-N atoms are *cis*. This is presumably due to the structural demands imparted by (pyrrole)_2_dien Schiff base ligand which has forced the [Co{(pyrrole)_2_dien}(4-Mepy)]^+^ to attain such a twisted structure.

### Experimental

The ligand was synthesized according to the literature (Kwiatkowski *et al.*, 1993). To a stirring solution of Co(CH_3_COO)_2_.4H_2_O (0.249 g, 1 mmol) in absolute ethanol (50 ml) was added an equimolar amount of ligand (0.256 g, 1 mmol). The pink solution turned brown immediately upon the formation of Co(III) complex. To this solution was added dropwise 7.5 mmol of the 4-methylpyridine. The reaction mixture was stirred for about 4 h and then filtered off. To the brown filtrate was added NaBPh_4_ (0.342 g, 1 mmol). The solution was left undisturbed for three days. The brown microcrystalline product was filtered off and washed with cold methanol. Brown crystals of the compound suitable for X-ray crystallography were obtained by diffusion of diethyl ether in to the acetone solution of the product.

### Refinement

All H atoms attached to C atoms were fixed geometrically and treated as riding with C—H = 0.95Å (aromatic), 0.99Å (methylene) and 0.98Å (methyl) with U_iso_(H) = 1.2 U_eq_(aromatic, methylene) or U_iso_(H) = 1.5 U_eq_(methyl). The N(3) bonded hydrogen H(3n) was freely refined.

### Crystal data

**Table:**

[Co(C_14_H_17_N_5_)(C_6_H_7_N)](C_24_H_20_B)	*F*(000) = 1528
*M**_r_* = 726.59	*D*_x_ = 1.306 Mg m^−^^3^
Monoclinic, *P*2_1_/*n*	Mo *K*α radiation, λ = 0.71073 Å
Hall symbol: -P 2yn	Cell parameters from 7791 reflections
*a* = 11.0332 (16) Å	θ = 2.4–30.0°
*b* = 19.559 (3) Å	µ = 0.51 mm^−^^1^
*c* = 17.138 (3) Å	*T* = 100 K
β = 92.164 (2)°	Prism, brown
*V* = 3695.7 (9) Å^3^	0.52 × 0.46 × 0.35 mm
*Z* = 4	

### Data collection

**Table:**

Bruker SMART APEX CCD diffractometer	10797 independent reflections
Radiation source: normal-focus sealed tube	8767 reflections with *I* > 2σ(*I*)
graphite	*R*_int_ = 0.036
\ and ω scans	θ_max_ = 30.0°, θ_min_ = 2.4°
Absorption correction: multi-scan (*SADABS*; Bruker, 2003)	*h* = −15→15
*T*_min_ = 0.71, *T*_max_ = 0.84	*k* = −27→27
66110 measured reflections	*l* = −24→24

### Refinement

**Table:**

Refinement on *F*^2^	Primary atom site location: structure-invariant direct methods
Least-squares matrix: full	Secondary atom site location: difference Fourier map
*R*[*F*^2^ > 2σ(*F*^2^)] = 0.038	Hydrogen site location: inferred from neighbouring sites
*wR*(*F*^2^) = 0.104	H atoms treated by a mixture of independent and constrained refinement
*S* = 1.07	*w* = 1/[σ^2^(*F*_o_^2^) + (0.0519*P*)^2^ + 1.7914*P*] where *P* = (*F*_o_^2^ + 2*F*_c_^2^)/3
10797 reflections	(Δ/σ)_max_ = 0.002
474 parameters	Δρ_max_ = 0.57 e Å^−^^3^
0 restraints	Δρ_min_ = −0.22 e Å^−^^3^

### Special details

**Table:**

Geometry. All esds (except the esd in the dihedral angle between two l.s. planes) are estimated using the full covariance matrix. The cell esds are taken into account individually in the estimation of esds in distances, angles and torsion angles; correlations between esds in cell parameters are only used when they are defined by crystal symmetry. An approximate (isotropic) treatment of cell esds is used for estimating esds involving l.s. planes.
Refinement. Refinement of *F*^2^ against ALL reflections. The weighted *R*-factor wR and goodness of fit *S* are based on *F*^2^, conventional *R*-factors *R* are based on *F*, with *F* set to zero for negative *F*^2^. The threshold expression of *F*^2^ > σ(*F*^2^) is used only for calculating *R*-factors(gt) etc. and is not relevant to the choice of reflections for refinement. *R*-factors based on *F*^2^ are statistically about twice as large as those based on *F*, and *R*- factors based on ALL data will be even larger.

### Fractional atomic coordinates and isotropic or equivalent isotropic displacement parameters (Å^2^)

**Table:**

	*x*	*y*	*z*	*U*_iso_*/*U*_eq_	
Co1	0.270495 (16)	0.246611 (9)	0.511104 (10)	0.01323 (5)	
N1	0.29289 (11)	0.26519 (6)	0.61977 (7)	0.0168 (2)	
N2	0.35020 (11)	0.16303 (6)	0.54069 (7)	0.0169 (2)	
N3	0.25046 (11)	0.19607 (6)	0.40752 (7)	0.0168 (2)	
H3N	0.2513 (16)	0.2203 (10)	0.3655 (11)	0.021 (4)*	
N4	0.11170 (11)	0.21273 (6)	0.52448 (7)	0.0174 (2)	
N5	0.18479 (10)	0.33239 (6)	0.49985 (7)	0.0168 (2)	
N6	0.42291 (10)	0.28990 (6)	0.48320 (7)	0.0149 (2)	
C1	0.26993 (15)	0.31506 (8)	0.67082 (9)	0.0243 (3)	
H1	0.2325	0.3575	0.6580	0.029*	
C2	0.30967 (17)	0.29492 (9)	0.74592 (10)	0.0324 (4)	
H2	0.3045	0.3212	0.7923	0.039*	
C3	0.35806 (16)	0.22953 (9)	0.74040 (9)	0.0281 (3)	
H3	0.3921	0.2025	0.7817	0.034*	
C4	0.34628 (13)	0.21183 (7)	0.66153 (8)	0.0190 (3)	
C5	0.37794 (13)	0.15587 (7)	0.61457 (8)	0.0192 (3)	
H5	0.4165	0.1161	0.6353	0.023*	
C6	0.37524 (14)	0.11217 (7)	0.48109 (9)	0.0208 (3)	
H6A	0.3190	0.0729	0.4845	0.025*	
H6B	0.4595	0.0952	0.4876	0.025*	
C7	0.35670 (14)	0.14869 (7)	0.40325 (8)	0.0204 (3)	
H7A	0.4305	0.1750	0.3914	0.024*	
H7B	0.3417	0.1148	0.3611	0.024*	
C8	0.13076 (14)	0.15881 (8)	0.40369 (9)	0.0218 (3)	
H8A	0.1404	0.1141	0.3775	0.026*	
H8B	0.0701	0.1859	0.3729	0.026*	
C9	0.08666 (14)	0.14765 (7)	0.48620 (9)	0.0216 (3)	
H9A	−0.0011	0.1371	0.4852	0.026*	
H9B	0.1318	0.1100	0.5128	0.026*	
C10	0.02770 (13)	0.25895 (7)	0.53121 (9)	0.0199 (3)	
H10	−0.0550	0.2476	0.5379	0.024*	
C11	0.06867 (13)	0.32712 (7)	0.52786 (9)	0.0192 (3)	
C12	0.02430 (14)	0.39236 (8)	0.54421 (9)	0.0240 (3)	
H12	−0.0528	0.4034	0.5636	0.029*	
C13	0.11611 (15)	0.43788 (8)	0.52628 (10)	0.0259 (3)	
H13	0.1133	0.4862	0.5311	0.031*	
C14	0.21353 (14)	0.39927 (7)	0.49984 (9)	0.0209 (3)	
H14	0.2886	0.4176	0.4842	0.025*	
C15	0.43211 (13)	0.31823 (7)	0.41192 (8)	0.0182 (3)	
H15	0.3629	0.3178	0.3773	0.022*	
C16	0.53711 (13)	0.34777 (7)	0.38701 (8)	0.0206 (3)	
H16	0.5396	0.3666	0.3360	0.025*	
C17	0.63943 (13)	0.34989 (8)	0.43680 (9)	0.0208 (3)	
C18	0.62879 (13)	0.32259 (8)	0.51101 (9)	0.0206 (3)	
H18	0.6958	0.3242	0.5475	0.025*	
C19	0.52091 (12)	0.29305 (7)	0.53198 (8)	0.0177 (3)	
H19	0.5159	0.2743	0.5829	0.021*	
C20	0.75545 (15)	0.38114 (10)	0.41104 (11)	0.0336 (4)	
H20A	0.7603	0.3765	0.3543	0.050*	
H20B	0.8244	0.3576	0.4368	0.050*	
H20C	0.7574	0.4297	0.4252	0.050*	
B1	0.79035 (13)	0.10736 (8)	0.68052 (9)	0.0149 (3)	
C21	0.66500 (12)	0.07260 (7)	0.71036 (8)	0.0168 (2)	
C22	0.62423 (14)	0.08440 (8)	0.78607 (9)	0.0240 (3)	
H22	0.6663	0.1168	0.8183	0.029*	
C23	0.52496 (15)	0.05059 (9)	0.81561 (10)	0.0292 (3)	
H23	0.5006	0.0601	0.8671	0.035*	
C24	0.46146 (14)	0.00298 (9)	0.76998 (11)	0.0292 (4)	
H24	0.3943	−0.0208	0.7901	0.035*	
C25	0.49725 (13)	−0.00932 (8)	0.69501 (10)	0.0251 (3)	
H25	0.4538	−0.0413	0.6630	0.030*	
C26	0.59709 (13)	0.02504 (7)	0.66600 (9)	0.0195 (3)	
H26	0.6198	0.0158	0.6141	0.023*	
C27	0.79551 (13)	0.18974 (7)	0.70191 (8)	0.0169 (3)	
C28	0.90322 (14)	0.22802 (8)	0.70670 (8)	0.0207 (3)	
H28	0.9785	0.2044	0.7061	0.025*	
C29	0.90444 (16)	0.29928 (8)	0.71231 (9)	0.0256 (3)	
H29	0.9796	0.3231	0.7147	0.031*	
C30	0.79626 (17)	0.33553 (8)	0.71446 (9)	0.0278 (3)	
H30	0.7966	0.3841	0.7168	0.033*	
C31	0.68804 (16)	0.29965 (8)	0.71308 (9)	0.0256 (3)	
H31	0.6134	0.3235	0.7161	0.031*	
C32	0.68832 (14)	0.22831 (8)	0.70729 (8)	0.0208 (3)	
H32	0.6129	0.2048	0.7070	0.025*	
C33	0.79120 (11)	0.10059 (7)	0.58466 (8)	0.0148 (2)	
C34	0.73321 (13)	0.14887 (7)	0.53542 (8)	0.0198 (3)	
H34	0.6976	0.1879	0.5583	0.024*	
C35	0.72561 (14)	0.14192 (8)	0.45425 (9)	0.0230 (3)	
H35	0.6856	0.1759	0.4231	0.028*	
C36	0.77648 (13)	0.08540 (8)	0.41903 (8)	0.0204 (3)	
H36	0.7714	0.0802	0.3639	0.025*	
C37	0.83468 (13)	0.03682 (7)	0.46558 (8)	0.0190 (3)	
H37	0.8704	−0.0019	0.4422	0.023*	
C38	0.84137 (12)	0.04421 (7)	0.54657 (8)	0.0167 (2)	
H38	0.8813	0.0099	0.5771	0.020*	
C39	0.90644 (12)	0.06510 (7)	0.71932 (8)	0.0167 (2)	
C40	1.02475 (13)	0.07448 (7)	0.69319 (8)	0.0191 (3)	
H40	1.0367	0.1059	0.6519	0.023*	
C41	1.12499 (13)	0.03983 (8)	0.72513 (9)	0.0231 (3)	
H41	1.2034	0.0485	0.7063	0.028*	
C42	1.11045 (15)	−0.00742 (8)	0.78447 (11)	0.0289 (3)	
H42	1.1784	−0.0314	0.8065	0.035*	
C43	0.99522 (16)	−0.01911 (8)	0.81106 (11)	0.0302 (4)	
H43	0.9838	−0.0517	0.8512	0.036*	
C44	0.89578 (14)	0.01675 (8)	0.77911 (9)	0.0230 (3)	
H44	0.8178	0.0081	0.7986	0.028*	

### Atomic displacement parameters (Å^2^)

**Table:**

	*U*^11^	*U*^22^	*U*^33^	*U*^12^	*U*^13^	*U*^23^
Co1	0.01395 (9)	0.01203 (9)	0.01370 (9)	0.00031 (6)	0.00058 (6)	0.00048 (6)
N1	0.0174 (5)	0.0172 (5)	0.0158 (5)	0.0008 (4)	0.0009 (4)	−0.0002 (4)
N2	0.0189 (5)	0.0131 (5)	0.0187 (6)	0.0015 (4)	0.0020 (4)	−0.0001 (4)
N3	0.0184 (5)	0.0158 (5)	0.0164 (5)	−0.0007 (4)	0.0005 (4)	0.0001 (4)
N4	0.0172 (5)	0.0167 (5)	0.0185 (6)	−0.0028 (4)	0.0022 (4)	0.0008 (4)
N5	0.0156 (5)	0.0159 (5)	0.0187 (6)	0.0016 (4)	−0.0004 (4)	0.0014 (4)
N6	0.0146 (5)	0.0142 (5)	0.0157 (5)	0.0006 (4)	−0.0002 (4)	0.0011 (4)
C1	0.0293 (8)	0.0226 (7)	0.0210 (7)	0.0034 (6)	0.0018 (6)	−0.0043 (6)
C2	0.0451 (10)	0.0344 (9)	0.0177 (7)	0.0057 (7)	0.0008 (7)	−0.0061 (6)
C3	0.0343 (9)	0.0332 (8)	0.0167 (7)	0.0035 (7)	−0.0012 (6)	0.0030 (6)
C4	0.0197 (6)	0.0201 (6)	0.0173 (6)	0.0004 (5)	0.0007 (5)	0.0032 (5)
C5	0.0194 (6)	0.0171 (6)	0.0210 (7)	0.0013 (5)	0.0016 (5)	0.0056 (5)
C6	0.0248 (7)	0.0143 (6)	0.0235 (7)	0.0035 (5)	0.0030 (5)	−0.0014 (5)
C7	0.0240 (7)	0.0179 (6)	0.0194 (7)	0.0035 (5)	0.0030 (5)	−0.0030 (5)
C8	0.0240 (7)	0.0205 (6)	0.0208 (7)	−0.0063 (5)	−0.0013 (5)	−0.0029 (5)
C9	0.0226 (7)	0.0177 (6)	0.0246 (7)	−0.0060 (5)	0.0018 (5)	−0.0003 (5)
C10	0.0155 (6)	0.0252 (7)	0.0190 (6)	−0.0004 (5)	0.0019 (5)	0.0005 (5)
C11	0.0157 (6)	0.0218 (7)	0.0203 (7)	0.0034 (5)	0.0004 (5)	0.0005 (5)
C12	0.0221 (7)	0.0244 (7)	0.0255 (7)	0.0083 (6)	−0.0003 (6)	−0.0013 (6)
C13	0.0296 (8)	0.0172 (6)	0.0305 (8)	0.0071 (6)	−0.0023 (6)	−0.0013 (6)
C14	0.0225 (7)	0.0161 (6)	0.0240 (7)	0.0016 (5)	−0.0010 (5)	0.0018 (5)
C15	0.0180 (6)	0.0192 (6)	0.0172 (6)	−0.0014 (5)	−0.0028 (5)	0.0035 (5)
C16	0.0207 (7)	0.0227 (7)	0.0182 (7)	−0.0035 (5)	−0.0004 (5)	0.0052 (5)
C17	0.0176 (6)	0.0223 (7)	0.0224 (7)	−0.0029 (5)	0.0008 (5)	0.0024 (5)
C18	0.0162 (6)	0.0243 (7)	0.0208 (7)	−0.0011 (5)	−0.0044 (5)	0.0022 (5)
C19	0.0180 (6)	0.0188 (6)	0.0160 (6)	0.0002 (5)	−0.0015 (5)	0.0016 (5)
C20	0.0207 (7)	0.0480 (10)	0.0320 (9)	−0.0113 (7)	−0.0003 (6)	0.0091 (8)
B1	0.0138 (6)	0.0163 (6)	0.0146 (7)	0.0003 (5)	0.0007 (5)	−0.0004 (5)
C21	0.0142 (6)	0.0181 (6)	0.0182 (6)	0.0031 (5)	0.0009 (5)	0.0025 (5)
C22	0.0208 (7)	0.0314 (8)	0.0199 (7)	0.0002 (6)	0.0025 (5)	−0.0002 (6)
C23	0.0219 (7)	0.0401 (9)	0.0261 (8)	0.0028 (6)	0.0087 (6)	0.0058 (7)
C24	0.0154 (7)	0.0309 (8)	0.0419 (10)	0.0010 (6)	0.0082 (6)	0.0116 (7)
C25	0.0162 (6)	0.0187 (6)	0.0403 (9)	0.0001 (5)	0.0010 (6)	0.0016 (6)
C26	0.0174 (6)	0.0166 (6)	0.0247 (7)	0.0013 (5)	0.0026 (5)	0.0003 (5)
C27	0.0204 (6)	0.0187 (6)	0.0115 (6)	0.0015 (5)	−0.0006 (5)	0.0001 (5)
C28	0.0236 (7)	0.0205 (6)	0.0179 (6)	−0.0011 (5)	0.0012 (5)	−0.0003 (5)
C29	0.0352 (8)	0.0212 (7)	0.0204 (7)	−0.0071 (6)	0.0008 (6)	−0.0006 (5)
C30	0.0485 (10)	0.0171 (7)	0.0174 (7)	0.0023 (6)	−0.0028 (6)	−0.0008 (5)
C31	0.0353 (8)	0.0242 (7)	0.0168 (7)	0.0110 (6)	−0.0039 (6)	−0.0033 (5)
C32	0.0236 (7)	0.0226 (7)	0.0160 (6)	0.0040 (5)	−0.0030 (5)	−0.0027 (5)
C33	0.0126 (5)	0.0170 (6)	0.0150 (6)	−0.0024 (4)	0.0006 (4)	−0.0005 (5)
C34	0.0211 (6)	0.0210 (6)	0.0171 (6)	0.0035 (5)	0.0000 (5)	−0.0006 (5)
C35	0.0260 (7)	0.0255 (7)	0.0172 (7)	0.0035 (6)	−0.0024 (5)	0.0022 (5)
C36	0.0231 (7)	0.0236 (7)	0.0146 (6)	−0.0040 (5)	0.0011 (5)	−0.0013 (5)
C37	0.0220 (7)	0.0169 (6)	0.0184 (6)	−0.0040 (5)	0.0029 (5)	−0.0028 (5)
C38	0.0182 (6)	0.0152 (6)	0.0166 (6)	−0.0024 (5)	0.0007 (5)	0.0002 (5)
C39	0.0167 (6)	0.0169 (6)	0.0165 (6)	0.0005 (5)	−0.0009 (5)	−0.0030 (5)
C40	0.0185 (6)	0.0229 (6)	0.0158 (6)	0.0016 (5)	0.0008 (5)	−0.0042 (5)
C41	0.0171 (6)	0.0262 (7)	0.0260 (7)	0.0042 (5)	0.0002 (5)	−0.0083 (6)
C42	0.0239 (7)	0.0239 (7)	0.0381 (9)	0.0074 (6)	−0.0083 (6)	−0.0011 (6)
C43	0.0297 (8)	0.0225 (7)	0.0379 (9)	0.0008 (6)	−0.0063 (7)	0.0104 (7)
C44	0.0201 (7)	0.0202 (7)	0.0284 (8)	−0.0010 (5)	−0.0027 (6)	0.0045 (6)

### Geometric parameters (Å, °)

**Table:**

Co1—N4	1.8952 (12)	C19—H19	0.9500
Co1—N1	1.9043 (12)	C20—H20A	0.9800
Co1—N2	1.9156 (12)	C20—H20B	0.9800
Co1—N5	1.9320 (12)	C20—H20C	0.9800
Co1—N6	1.9581 (12)	B1—C21	1.640 (2)
Co1—N3	2.0367 (12)	B1—C39	1.644 (2)
N1—C1	1.3408 (18)	B1—C33	1.649 (2)
N1—C4	1.3845 (18)	B1—C27	1.653 (2)
N2—C5	1.2990 (19)	C21—C26	1.401 (2)
N2—C6	1.4595 (18)	C21—C22	1.408 (2)
N3—C7	1.4982 (18)	C22—C23	1.391 (2)
N3—C8	1.5077 (18)	C22—H22	0.9500
N3—H3N	0.86 (2)	C23—C24	1.389 (3)
N4—C10	1.3030 (18)	C23—H23	0.9500
N4—C9	1.4537 (18)	C24—C25	1.379 (2)
N5—C14	1.3460 (18)	C24—H24	0.9500
N5—C11	1.3888 (18)	C25—C26	1.398 (2)
N6—C19	1.3429 (17)	C25—H25	0.9500
N6—C15	1.3488 (17)	C26—H26	0.9500
C1—C2	1.400 (2)	C27—C28	1.404 (2)
C1—H1	0.9500	C27—C32	1.409 (2)
C2—C3	1.391 (2)	C28—C29	1.397 (2)
C2—H2	0.9500	C28—H28	0.9500
C3—C4	1.397 (2)	C29—C30	1.390 (2)
C3—H3	0.9500	C29—H29	0.9500
C4—C5	1.410 (2)	C30—C31	1.384 (2)
C5—H5	0.9500	C30—H30	0.9500
C6—C7	1.520 (2)	C31—C32	1.399 (2)
C6—H6A	0.9900	C31—H31	0.9500
C6—H6B	0.9900	C32—H32	0.9500
C7—H7A	0.9900	C33—C34	1.4044 (19)
C7—H7B	0.9900	C33—C38	1.4055 (18)
C8—C9	1.528 (2)	C34—C35	1.397 (2)
C8—H8A	0.9900	C34—H34	0.9500
C8—H8B	0.9900	C35—C36	1.388 (2)
C9—H9A	0.9900	C35—H35	0.9500
C9—H9B	0.9900	C36—C37	1.383 (2)
C10—C11	1.410 (2)	C36—H36	0.9500
C10—H10	0.9500	C37—C38	1.3948 (19)
C11—C12	1.399 (2)	C37—H37	0.9500
C12—C13	1.392 (2)	C38—H38	0.9500
C12—H12	0.9500	C39—C44	1.403 (2)
C13—C14	1.403 (2)	C39—C40	1.4079 (19)
C13—H13	0.9500	C40—C41	1.392 (2)
C14—H14	0.9500	C40—H40	0.9500
C15—C16	1.3768 (19)	C41—C42	1.388 (2)
C15—H15	0.9500	C41—H41	0.9500
C16—C17	1.390 (2)	C42—C43	1.386 (2)
C16—H16	0.9500	C42—H42	0.9500
C17—C18	1.388 (2)	C43—C44	1.397 (2)
C17—C20	1.500 (2)	C43—H43	0.9500
C18—C19	1.383 (2)	C44—H44	0.9500
C18—H18	0.9500		
			
N4—Co1—N1	92.00 (5)	C17—C16—H16	120.1
N4—Co1—N2	94.98 (5)	C18—C17—C16	117.14 (13)
N1—Co1—N2	82.11 (5)	C18—C17—C20	121.94 (14)
N4—Co1—N5	82.22 (5)	C16—C17—C20	120.92 (14)
N1—Co1—N5	88.67 (5)	C19—C18—C17	120.15 (13)
N2—Co1—N5	170.28 (5)	C19—C18—H18	119.9
N4—Co1—N6	170.80 (5)	C17—C18—H18	119.9
N1—Co1—N6	94.32 (5)	N6—C19—C18	122.55 (13)
N2—Co1—N6	92.51 (5)	N6—C19—H19	118.7
N5—Co1—N6	91.24 (5)	C18—C19—H19	118.7
N4—Co1—N3	82.26 (5)	C17—C20—H20A	109.5
N1—Co1—N3	161.96 (5)	C17—C20—H20B	109.5
N2—Co1—N3	81.38 (5)	H20A—C20—H20B	109.5
N5—Co1—N3	107.33 (5)	C17—C20—H20C	109.5
N6—Co1—N3	93.61 (5)	H20A—C20—H20C	109.5
C1—N1—C4	107.35 (12)	H20B—C20—H20C	109.5
C1—N1—Co1	139.14 (11)	C21—B1—C39	108.60 (11)
C4—N1—Co1	113.49 (9)	C21—B1—C33	108.30 (11)
C5—N2—C6	124.39 (12)	C39—B1—C33	109.21 (11)
C5—N2—Co1	116.03 (10)	C21—B1—C27	110.93 (11)
C6—N2—Co1	119.57 (9)	C39—B1—C27	112.38 (11)
C7—N3—C8	112.61 (11)	C33—B1—C27	107.33 (11)
C7—N3—Co1	106.46 (9)	C26—C21—C22	115.21 (13)
C8—N3—Co1	109.79 (9)	C26—C21—B1	123.11 (12)
C7—N3—H3N	105.5 (12)	C22—C21—B1	121.45 (13)
C8—N3—H3N	105.4 (12)	C23—C22—C21	122.73 (15)
Co1—N3—H3N	117.2 (13)	C23—C22—H22	118.6
C10—N4—C9	121.58 (13)	C21—C22—H22	118.6
C10—N4—Co1	115.59 (10)	C24—C23—C22	120.09 (15)
C9—N4—Co1	114.36 (9)	C24—C23—H23	120.0
C14—N5—C11	107.02 (12)	C22—C23—H23	120.0
C14—N5—Co1	136.85 (10)	C25—C24—C23	119.04 (14)
C11—N5—Co1	110.82 (9)	C25—C24—H24	120.5
C19—N6—C15	117.30 (12)	C23—C24—H24	120.5
C19—N6—Co1	123.13 (9)	C24—C25—C26	120.30 (15)
C15—N6—Co1	119.57 (9)	C24—C25—H25	119.8
N1—C1—C2	109.56 (14)	C26—C25—H25	119.8
N1—C1—H1	125.2	C25—C26—C21	122.61 (14)
C2—C1—H1	125.2	C25—C26—H26	118.7
C3—C2—C1	107.70 (14)	C21—C26—H26	118.7
C3—C2—H2	126.2	C28—C27—C32	114.92 (13)
C1—C2—H2	126.2	C28—C27—B1	123.68 (12)
C2—C3—C4	105.77 (14)	C32—C27—B1	120.97 (12)
C2—C3—H3	127.1	C29—C28—C27	122.81 (14)
C4—C3—H3	127.1	C29—C28—H28	118.6
N1—C4—C3	109.61 (13)	C27—C28—H28	118.6
N1—C4—C5	113.57 (13)	C30—C29—C28	120.31 (15)
C3—C4—C5	136.77 (14)	C30—C29—H29	119.8
N2—C5—C4	114.73 (13)	C28—C29—H29	119.8
N2—C5—H5	122.6	C31—C30—C29	118.81 (14)
C4—C5—H5	122.6	C31—C30—H30	120.6
N2—C6—C7	105.73 (11)	C29—C30—H30	120.6
N2—C6—H6A	110.6	C30—C31—C32	120.17 (15)
C7—C6—H6A	110.6	C30—C31—H31	119.9
N2—C6—H6B	110.6	C32—C31—H31	119.9
C7—C6—H6B	110.6	C31—C32—C27	122.88 (15)
H6A—C6—H6B	108.7	C31—C32—H32	118.6
N3—C7—C6	109.11 (11)	C27—C32—H32	118.6
N3—C7—H7A	109.9	C34—C33—C38	115.27 (12)
C6—C7—H7A	109.9	C34—C33—B1	121.68 (12)
N3—C7—H7B	109.9	C38—C33—B1	122.88 (12)
C6—C7—H7B	109.9	C35—C34—C33	122.80 (13)
H7A—C7—H7B	108.3	C35—C34—H34	118.6
N3—C8—C9	109.74 (12)	C33—C34—H34	118.6
N3—C8—H8A	109.7	C36—C35—C34	120.02 (14)
C9—C8—H8A	109.7	C36—C35—H35	120.0
N3—C8—H8B	109.7	C34—C35—H35	120.0
C9—C8—H8B	109.7	C37—C36—C35	118.88 (13)
H8A—C8—H8B	108.2	C37—C36—H36	120.6
N4—C9—C8	103.34 (11)	C35—C36—H36	120.6
N4—C9—H9A	111.1	C36—C37—C38	120.58 (13)
C8—C9—H9A	111.1	C36—C37—H37	119.7
N4—C9—H9B	111.1	C38—C37—H37	119.7
C8—C9—H9B	111.1	C37—C38—C33	122.45 (13)
H9A—C9—H9B	109.1	C37—C38—H38	118.8
N4—C10—C11	114.98 (13)	C33—C38—H38	118.8
N4—C10—H10	122.5	C44—C39—C40	115.12 (13)
C11—C10—H10	122.5	C44—C39—B1	123.30 (12)
N5—C11—C12	109.62 (13)	C40—C39—B1	121.57 (12)
N5—C11—C10	112.69 (12)	C41—C40—C39	122.96 (14)
C12—C11—C10	137.68 (14)	C41—C40—H40	118.5
C13—C12—C11	106.03 (13)	C39—C40—H40	118.5
C13—C12—H12	127.0	C42—C41—C40	120.02 (14)
C11—C12—H12	127.0	C42—C41—H41	120.0
C12—C13—C14	107.46 (13)	C40—C41—H41	120.0
C12—C13—H13	126.3	C43—C42—C41	118.93 (14)
C14—C13—H13	126.3	C43—C42—H42	120.5
N5—C14—C13	109.86 (13)	C41—C42—H42	120.5
N5—C14—H14	125.1	C42—C43—C44	120.34 (15)
C13—C14—H14	125.1	C42—C43—H43	119.8
N6—C15—C16	123.10 (13)	C44—C43—H43	119.8
N6—C15—H15	118.4	C43—C44—C39	122.60 (14)
C16—C15—H15	118.4	C43—C44—H44	118.7
C15—C16—C17	119.72 (13)	C39—C44—H44	118.7
C15—C16—H16	120.1		
			
N4—Co1—N1—C1	−85.68 (16)	C14—N5—C11—C12	0.99 (17)
N2—Co1—N1—C1	179.57 (17)	Co1—N5—C11—C12	159.84 (10)
N5—Co1—N1—C1	−3.51 (16)	C14—N5—C11—C10	−179.68 (13)
N6—Co1—N1—C1	87.63 (16)	Co1—N5—C11—C10	−20.82 (15)
N3—Co1—N1—C1	−156.52 (16)	N4—C10—C11—N5	14.25 (19)
N4—Co1—N1—C4	92.32 (10)	N4—C10—C11—C12	−166.68 (17)
N2—Co1—N1—C4	−2.43 (10)	N5—C11—C12—C13	−0.50 (17)
N5—Co1—N1—C4	174.49 (10)	C10—C11—C12—C13	−179.59 (18)
N6—Co1—N1—C4	−94.37 (10)	C11—C12—C13—C14	−0.16 (18)
N3—Co1—N1—C4	21.5 (2)	C11—N5—C14—C13	−1.09 (17)
N4—Co1—N2—C5	−89.44 (11)	Co1—N5—C14—C13	−151.55 (12)
N1—Co1—N2—C5	1.90 (10)	C12—C13—C14—N5	0.79 (18)
N6—Co1—N2—C5	95.92 (11)	C19—N6—C15—C16	2.0 (2)
N3—Co1—N2—C5	−170.81 (11)	Co1—N6—C15—C16	−177.98 (11)
N4—Co1—N2—C6	90.25 (11)	N6—C15—C16—C17	−0.8 (2)
N1—Co1—N2—C6	−178.42 (11)	C15—C16—C17—C18	−1.2 (2)
N6—Co1—N2—C6	−84.40 (11)	C15—C16—C17—C20	179.41 (15)
N3—Co1—N2—C6	8.88 (10)	C16—C17—C18—C19	1.9 (2)
N4—Co1—N3—C7	−125.83 (9)	C20—C17—C18—C19	−178.75 (15)
N1—Co1—N3—C7	−53.5 (2)	C15—N6—C19—C18	−1.3 (2)
N2—Co1—N3—C7	−29.56 (9)	Co1—N6—C19—C18	178.70 (11)
N5—Co1—N3—C7	154.86 (9)	C17—C18—C19—N6	−0.7 (2)
N6—Co1—N3—C7	62.43 (9)	C39—B1—C21—C26	−97.53 (15)
N4—Co1—N3—C8	−3.66 (9)	C33—B1—C21—C26	20.96 (17)
N1—Co1—N3—C8	68.66 (19)	C27—B1—C21—C26	138.50 (13)
N2—Co1—N3—C8	92.61 (10)	C39—B1—C21—C22	76.73 (16)
N5—Co1—N3—C8	−82.97 (10)	C33—B1—C21—C22	−164.78 (13)
N6—Co1—N3—C8	−175.40 (9)	C27—B1—C21—C22	−47.24 (17)
N1—Co1—N4—C10	79.29 (11)	C26—C21—C22—C23	1.0 (2)
N2—Co1—N4—C10	161.54 (11)	B1—C21—C22—C23	−173.64 (14)
N5—Co1—N4—C10	−9.10 (11)	C21—C22—C23—C24	−0.1 (2)
N3—Co1—N4—C10	−117.88 (11)	C22—C23—C24—C25	−0.9 (2)
N1—Co1—N4—C9	−132.03 (10)	C23—C24—C25—C26	0.9 (2)
N2—Co1—N4—C9	−49.77 (10)	C24—C25—C26—C21	0.2 (2)
N5—Co1—N4—C9	139.59 (10)	C22—C21—C26—C25	−1.1 (2)
N3—Co1—N4—C9	30.80 (10)	B1—C21—C26—C25	173.48 (13)
N4—Co1—N5—C14	166.04 (16)	C21—B1—C27—C28	159.16 (13)
N1—Co1—N5—C14	73.84 (15)	C39—B1—C27—C28	37.37 (18)
N6—Co1—N5—C14	−20.45 (15)	C33—B1—C27—C28	−82.71 (15)
N3—Co1—N5—C14	−114.62 (15)	C21—B1—C27—C32	−28.74 (17)
N4—Co1—N5—C11	16.33 (10)	C39—B1—C27—C32	−150.53 (13)
N1—Co1—N5—C11	−75.87 (10)	C33—B1—C27—C32	89.39 (14)
N6—Co1—N5—C11	−170.16 (10)	C32—C27—C28—C29	−3.2 (2)
N3—Co1—N5—C11	95.66 (10)	B1—C27—C28—C29	169.37 (13)
N1—Co1—N6—C19	28.99 (11)	C27—C28—C29—C30	0.9 (2)
N2—Co1—N6—C19	−53.28 (11)	C28—C29—C30—C31	1.7 (2)
N5—Co1—N6—C19	117.75 (11)	C29—C30—C31—C32	−1.9 (2)
N3—Co1—N6—C19	−134.79 (11)	C30—C31—C32—C27	−0.6 (2)
N1—Co1—N6—C15	−151.02 (11)	C28—C27—C32—C31	3.0 (2)
N2—Co1—N6—C15	126.70 (11)	B1—C27—C32—C31	−169.73 (13)
N5—Co1—N6—C15	−62.27 (11)	C21—B1—C33—C34	85.08 (15)
N3—Co1—N6—C15	45.19 (11)	C39—B1—C33—C34	−156.82 (12)
C4—N1—C1—C2	0.81 (18)	C27—B1—C33—C34	−34.75 (16)
Co1—N1—C1—C2	178.89 (13)	C21—B1—C33—C38	−90.05 (14)
N1—C1—C2—C3	−0.6 (2)	C39—B1—C33—C38	28.05 (17)
C1—C2—C3—C4	0.1 (2)	C27—B1—C33—C38	150.13 (12)
C1—N1—C4—C3	−0.77 (17)	C38—C33—C34—C35	−0.1 (2)
Co1—N1—C4—C3	−179.40 (11)	B1—C33—C34—C35	−175.60 (13)
C1—N1—C4—C5	−178.76 (13)	C33—C34—C35—C36	0.1 (2)
Co1—N1—C4—C5	2.61 (15)	C34—C35—C36—C37	−0.3 (2)
C2—C3—C4—N1	0.43 (19)	C35—C36—C37—C38	0.5 (2)
C2—C3—C4—C5	177.73 (18)	C36—C37—C38—C33	−0.5 (2)
C6—N2—C5—C4	179.39 (13)	C34—C33—C38—C37	0.32 (19)
Co1—N2—C5—C4	−0.94 (16)	B1—C33—C38—C37	175.74 (12)
N1—C4—C5—N2	−1.10 (19)	C21—B1—C39—C44	−11.12 (18)
C3—C4—C5—N2	−178.34 (17)	C33—B1—C39—C44	−129.03 (14)
C5—N2—C6—C7	−166.34 (13)	C27—B1—C39—C44	111.99 (15)
Co1—N2—C6—C7	14.00 (15)	C21—B1—C39—C40	167.74 (12)
C8—N3—C7—C6	−74.96 (14)	C33—B1—C39—C40	49.83 (16)
Co1—N3—C7—C6	45.40 (13)	C27—B1—C39—C40	−69.15 (16)
N2—C6—C7—N3	−38.32 (15)	C44—C39—C40—C41	−1.5 (2)
C7—N3—C8—C9	96.87 (14)	B1—C39—C40—C41	179.59 (13)
Co1—N3—C8—C9	−21.57 (14)	C39—C40—C41—C42	1.3 (2)
C10—N4—C9—C8	97.92 (15)	C40—C41—C42—C43	−0.1 (2)
Co1—N4—C9—C8	−48.69 (13)	C41—C42—C43—C44	−0.7 (3)
N3—C8—C9—N4	42.79 (15)	C42—C43—C44—C39	0.5 (3)
C9—N4—C10—C11	−146.56 (13)	C40—C39—C44—C43	0.6 (2)
Co1—N4—C10—C11	−0.32 (17)	B1—C39—C44—C43	179.51 (14)

## References

[bb1] Amirnasr, M., Schenk, K. J., Meghdadi, S. & Morshedi, M. (2006). *Polyhedron*, **25**, 671–677.

[bb2] Bruker (2003). *SMART*, *SAINT*, *SADABS* and *XPREP* Bruker AXS Inc., Madison, Wisconsin, USA.

[bb3] Kwiatkowski, E., Kwiatkowski, M., Olechnowicz, A. & Bandoli, G. (1993). *J. Crystallogr. Spectrosc. Res.* **23**, 423–429.

[bb4] Meghdadi, S., Daran, J.-C., Amirnasr, M. & Morshedi, M. (2007). *Acta Cryst.* E**63**, m982–m984.

[bb5] Meghdadi, S., Schenk, K. J., Amirnasr, M. & Fadaee, F. (2008). *Acta Cryst.* E**64**, m479–m480. 10.1107/S1600536808003875 PMC2960776 21201867

[bb6] Miodragović, D. U., Bogdanović, G. A., Miodragović, Z. M., Radulović, M. D., Novaković, S. B., Kaluderović, G. N. & Kozlowski, H. (2006). *J. Inorg. Biochem.* **100**, 1568–1574. 10.1016/j.jinorgbio.2006.05.009 16831463

[bb7] Mishra, A., Kaushik, N. K., Verma, A. K. & Gupta, R. (2008). *Eur. J. Med. Chem* **43**, 2189–2196. 10.1016/j.ejmech.2007.08.015 17959275

[bb8] Morshedi, M., Meghdadi, S. & Schenk, K. J. (2006). *Acta Cryst.* C**62**, m87–m89. 10.1107/S0108270105043258 16518037

[bb9] Nishinaga, A. & Tomita, H. (1980). *J. Mol. Catal.* **7**, 179–199.

[bb10] Park, S., Mathur, V. K. & Planalp, R. P. (1998). *Polyhedron*, **17**, 325–330.

[bb11] Sheldrick, G. M. (2008). *Acta Cryst.* A**64**, 112–122. 10.1107/S0108767307043930 18156677

[bb12] Speiser, B. & Stahl, H. (1995). *Angew. Chem. Int. Ed.* **34**, 1086–1089.

